# Focal adhesion kinase regulates the activity of the osmosensitive transcription factor TonEBP/NFAT5 under hypertonic conditions

**DOI:** 10.3389/fphys.2014.00123

**Published:** 2014-04-04

**Authors:** Wolfgang Neuhofer, Christoph Küper, Julia Lichtnekert, Konstantin Holzapfel, Khader V. Rupanagudi, Maria-Luisa Fraek, Helmut Bartels, Franz-Xaver Beck

**Affiliations:** ^1^Division of Nephrology, Medical Clinic and Policlinic IV, University of MunichMunich, Germany; ^2^Department of Cellular Physiology, University of MunichMunich, Germany; ^3^Department of Radiology, Klinikum Rechts der Isar, Technical University of MunichMunich, Germany; ^4^Center of Anatomy, Institute of Functional and Applied Anatomy, Hannover Medical SchoolHannover, Germany

**Keywords:** TonEBP/NFAT5, osmotic stress, focal adhesion kinase, gene regulation, renal medulla

## Abstract

TonEBP/NFAT5 is a major regulator of the urinary concentrating process and is essential for the osmoadaptation of renal medullary cells. Focal adhesion kinase (FAK) is a mechanosensitive non-receptor protein tyrosine kinase expressed abundantly in the renal medulla. Since osmotic stress causes cell shrinkage, the present study investigated the contribution of FAK on TonEBP/NFAT5 activation. Osmotic stress induced time-dependent activation of FAK as evidenced by phosphorylation at Tyr-397, and furosemide reduces FAK Tyr-397 phosphorylation in the rat renal medulla. Both pharmacological inhibition of FAK and siRNA-mediated knockdown of FAK drastically reduced TonEBP/NFAT5 transcriptional activity and target gene expression in HEK293 cells. This effect was not mediated by impaired nuclear translocation or by reduced transactivating activity of TonEBP/NFAT5. However, TonEBP/NFAT5 abundance under hypertonic conditions was diminished by 50% by FAK inhibition or siRNA knockdown of FAK. FAK inhibition only marginally reduced transcription of the TonEBP/NFAT5 gene. Rather, TonEBP/NFAT5 mRNA stability was diminished significantly by FAK inhibition, which correlated with reduced reporter activity of the TonEBP/NFAT5 mRNA 3′ untranslated region (3′-UTR). In conclusion, FAK is a major regulator of TonEBP/NFAT5 activity by increasing its abundance via stabilization of the mRNA. This in turn, depends on the presence of the TonEBP/NFAT5 3′-UTR.

## Introduction

Tonicity-responsive enhancer binding protein/nuclear factor of activated T cells 5 (TonEBP/NFAT5) is an osmosensitive transcription factor necessary for the urinary concentrating process and osmoadaptation of renal medullary cells (Miyakawa et al., 1999). In the kidney medulla, TonEBP/NFAT5 stimulates the expression of essential components of the urinary concentrating mechanism, including NKCC2, UT-A1, and ClC-K1 with its functional subunit Barttin, which are required for generating the corticomedullary osmotic gradient (Neuhofer and Beck, 2005; Fenton and Knepper, 2007; Küper et al., 2012b). In addition, aquaporin-2 (AQP-2) water channels are essential for water reabsorption along the collecting duct and, hence, production of concentrated urine (Fenton and Knepper, 2007; Küper et al., 2012b). As a consequence of the operation of the urinary concentrating mechanism, renal medullary cells are exposed to extracellular osmolalities several fold higher than in the systemic circulation. Remarkably, TonEBP/NFAT5 in parallel stimulates the expression of various osmoprotective genes that mediate the intracellular accumulation of high amounts of compatible organic osmolytes and specific heat shock proteins (Neuhofer and Beck, 2005; Jeon et al., 2006). The enhanced expression of these osmosensitive genes requires the interaction of TonEBP/NFAT5 with tonicity-responsive enhancer elements (TonE) in the promoter region of the respective target genes (Neuhofer and Beck, 2005; Jeon et al., 2006). In addition, recent evidence suggests that TonEBP/NFAT5 is critically involved in the expression of various proinflammatory cytokines in cells exposed to local hypertonicity, as present under pathophysiological conditions (Machnik et al., 2009; Neuhofer, 2010; Küper et al., 2012a).

Cells exposed to elevated extracellular solute concentrations in the form of NaCl initially shrink because NaCl is functionally excluded from the intracellular space by the action of the Na-K-ATPase (Neuhofer and Beck, 2005; Jeon et al., 2006). It has been proposed that changes in cell volume following alterations in extracellular tonicity are important initiators of signaling events in the initial phase of osmotic stress (Zhang et al., 1998; Hoffmann et al., 2009). Cell shrinkage affects cell/cell and cell/matrix interactions, which are transduced into intracellular signals by integrins. Integrins play critical roles in normal physiology and pathology, since they regulate important cell functions, including adhesion, shape, polarity, growth, differentiation, and motility (Pozzi and Zent, 2003; Hsia et al., 2005; Mitra et al., 2005). Integrins, transmembrane receptors for extracellular matrix (ECM) components, interact with ECM proteins via their extracellular domains, while their cytoplasmic tail plays a pivotal role in mediating integrin-dependent cellular functions. The cytoplasmic domain of integrins interacts with the cytoskeleton, signaling molecules and other cellular proteins, resulting in the regulation of diverse biological processes (Pozzi and Zent, 2003; Hsia et al., 2005; Mitra and Schlaepfer, 2006).

Focal adhesion kinase (FAK) is a widely expressed non-receptor protein tyrosine kinase (PTK) that is intimately involved in integrin-mediated signal transduction and is a central signaling component downstream of integrins (Schlaepfer et al., 1994; Mitra and Schlaepfer, 2006). In response to integrin engagement, FAK is autophosphorylated at Tyr-397, which entails diverse intracellular signaling events (Schlaepfer and Hunter, 1997). Since FAK is a mechanosensitive kinase, this non-receptor PTK could be activated by osmotic stress and could contribute to the activation of TonEBP/NFAT5 under hypertonic conditions. Thus, the present study addressed this hypothesis using a cell culture model and delineated the molecular mechanisms.

## Methods

### Materials

The specific pharmacological FAK inhibitor PF-228 was obtained from Tocris Bioscience (Bristol, UK), anti-TonEBP/NFAT5 and anti-phospho-FAK Tyr-397 from Santa Cruz Biotechnology (Santa, CA, USA) and anti-FAK from BD Bioscience (Heidelberg, Germany). Anti-HSP70 was purchased from Biomol (Hamburg, Germany), anti-β actin was obtained from Sigma and horseradish peroxidase-conjugated anti-rabbit IgG from Jackson ImmunoResearch (West Grove, PA, USA). Unless otherwise indicated, other reagents were purchased from Biomol (Hamburg, Germany), Biozol (Eching, Germany), Carl Roth (Karlsruhe, Germany), or Sigma (Taufkirchen, Germany).

### Animal experiments

All experiments were conducted in accordance with German federal laws relating to animal experimentation. Male Wistar rats (~250 g; Charles River, Sulzfeld, Germany) with free access to standard chow and tap water received an intravenous injection of furosemide (20 mg/kg bw) or the same volume of phosphate-buffered saline (PBS). After 20 and 40 min, the kidneys were removed and immediately dissected on ice into cortex, outer and inner medulla, and processed for immunohistochemistry and Western blotting, respectively.

### Immunohistochemistry

Immunohistochemistry for FAK was performed on kidneys obtained from Male Wistar rats (~250 g). Using a polyclonal FAK antibody (1:100 dilution in PBS containing 1% BSA), FAK was immunolocalized on 5-μm-thick, formalin-fixed, paraffin-embedded tissue sections containing all kidney zones from cortex to papilla. After incubation with biotin-conjugated secondary antibody, the immunocomplexes were visualized using an avidin-biotin complex detection kit (Vectastain ABC Kit; Vector Laboratories, Burlingame, CA, USA) and counterstained with hematoxylin. Details of the procedure are described elsewhere (Holzapfel et al., 2007).

### Cell culture

Human embryonic kidney (HEK) 293 cells (CRL-1573), Madin-Darby canine kidney cells (MDCK; CCL-34), mouse mesangial cells (MSG; CRL1927), FAK-deficient mouse embryonic fibroblasts (MEF^FAK−/−^), and control MEF (MEF^FAK+/+^) were obtained from the American Type Culture Collection (ATCC, Manassas, VA, USA). The cells were cultured in standard DMEM (MDCK, MEF) or high-glucose DMEM (HEK293, MSG) supplemented with 10% fetal bovine serum (Biochrom, Berlin, Germany), 100 U/ml penicillin and 100 μg/ml streptomycin (Invitrogen, Karlsruhe, Germany) at 37°C in a humidified atmosphere (5% CO_2_-95% air). In experiments with PF-228, the cells were preincubated with the compound or vehicle for 30 min prior to the experimental treatment. Medium osmolality was increased by dropwise addition of the required volume of a 4 M NaCl stock solution.

### Establishment of stable TonE reporter cell lines

Exponentially growing HEK293, MDCK, and MSG cells were cotransfected with pSEAP-TonE and pcDNA3.1 containing a neomycin-resistance cassette (Invitrogen, Karlsruhe, Germany) at a ratio of 20:1 (20 μg plasmid DNA/100-mm dish) using Metafectene (Biontex) as described previously (Küper et al., 2012b). One day after transfection, G418 was added (400 μg/ml for HEK293 and MSG cells, 600 μg/ml for MDCK cells), and G418-resistant clones expanded, screened for tonicity-inducible reporter activity, pooled and used for experiments.

### Reporter gene assays

pSEAP-TonE contains the secreted alkaline phosphatase (SEAP) ORF under control of two TonE elements as described (Küper et al., 2012b). The pLightswitch-TonEBP/NFAT5-Luc reporter plasmid contains 905 bp of the human TonEBP/NFAT5 promoter region upstream from the *Renilla reniformis* luciferase gene (Switch Gear Genomics; Menlo Park, CA, USA). The nucleotide sequence is available at http://switchdb.switchgeargenomics.com/productinfo/id_708254 and was added as supplementary file. The TonEBP/NFAT5-3′-UTR-Luc reporter vector was a kind gift of Dr. J. Ferraris (National Institutes of Health, Bethesda, MD, USA; Cai et al., 2005). It contains the *Photinus pyralis* luciferase gene upstream from the complete TonEBP/NFAT5-3′-UTR (bp 5905–14,219).

HEK293 cells were grown to ~80% confluency and transfected with the respective reporter constructs using Metafectene pro reagent (Biontex, Martinsried, Germany). After reaching confluency, the cells were treated as indicated and SEAP activity in the medium determined as described previously (Küper et al., 2012b). Luciferase activity was determined by the Luciferase Assay System (Promega, Madison, WI, USA) according to the manufacturer's recommendations using a Varian Cary Eclipse Fluorescence Spectrophotometer/Luminometer (Agilent Technologies, Santa Clara, CA, USA). For control of transfection efficiency, the cells were cotransfected with pcDNA3-lacZ, and β-galactosidase activity was determined as described previously (Küper et al., 2012b). Finally, luciferase activity was normalized to β-galactosidase activity.

### qRT-PCR analysis

For determination of mRNA expression levels, total RNA was recovered using TriFast Reagent (Peqlab, Erlangen, Germany) according to the manufacturer's recommendations. The primers (Metabion, Martinsried, Germany) used in these experiments were: Aldose reductase (AR)_fw: 5′-ATC GCA GCC AAG CAC AAT AA-3′; AR_rev: 5′-AGC AAT GCG TTC TGG TGT CA-3′; TonEBP/NFAT5_fw: 5′-AAT CGC CCA AGT CCC TCT AC-3′; TonEBP/NFAT5_rev: 5′-GGT GGT AAA GGA GCT GCA AG -3′; actin_fw: 5′- CCA ACC GCG AGA AGA TGA-3′; actin_rev: 5′- CCA GAG GCG TAC AGG GAT AG -3′. Experiments were performed on a Roche LightCycler 480, using the SensiMix SYBR One-Step Kit (Bioline, Luckenwalde, Germany) according to the manufacturer's recommendations. Relative mRNA expression of the respective genes was calculated by the 2^−ΔΔ*CT*^–method (Livak and Schmittgen, 2001), using β-actin as housekeeping gene. Specificity of PCR productformation was confirmed by monitoring melting point analysisand by agarose gel electrophoresis as described (Küper et al., 2012b).

### Western blot analysis

Following the experiments, the cells were washed three times with chilled PBS and lysed by the addition of 8 M urea/PBS (200 μl/100 mm dish). Mouse kidney specimens were homogenized in 8 M urea/PBS (100 μl/10 mg tissue) using a Potter-Elvehjem homogenizer. The lysates were cleared by centrifugation at 13,000 g at 4°C for 15 min, and the supernatant was used for analyses. Aliquots (30 μg protein) were subjected to 10% SDS-PAGE and blotted onto nitrocellulose membranes (Amersham Pharmacia Biotech, Buckinghamshire, UK). Non-specific binding sites were blocked with 5% non-fat dry milk in PBS containing 0.1% Tween 20 (PBS-T) and the membranes were subsequently incubated with primary antibodies in PBS-T containing 5% non-fat dry milk overnight at 4°C with agitation. Thereafter, the blots were washed three times with PBS-T for 5 min each and incubated with appropriate secondary antibodies at room temperature for 1 h in PBS-T containing 5% non-fat dry milk. After washing with PBS-T three times for 5 min each, immunocomplexes were visualized by enhanced chemiluminescence (Pierce, Rockford, IL, USA).

### Determination of TonEBP/NFAT5 transactivating activity

TonEBP/NFAT5 transactivating activity was determined using the Gal4 binary assay as described previously (Ferraris et al., 2002; Küper et al., 2012b). Gal4-TonEBP-TAD contains the yeast Gal4 DNA binding domain fused in-frame to the transactivation domain (TAD) of TonEBP/NFAT5 (amino acids 548-1531; kindly provided by Dr. J. Ferraris). pFR-SEAP contains five tandem repeats of the Gal4 binding site upstream of a minimal promoter and the SEAP ORF (Stratagene, Amsterdam, Netherlands). Briefly, 4 × 10^6^ cells were transfected by electroporation with 20 μg pGal4-TonEBP-TAD and 20 μg pFR-SEAP (350 V, 950 μF) using a Bio-Rad Genepulser Xcell apparatus (Bio-Rad, Hercules, CA), and subsequently plated into 4–6 wells of a 24-well plate. After 24–48 h, the cells were treated as indicated and SEAP activity in the medium was determined as described above.

### Small interfering RNA knockdown of FAK

Accell SMARTpool small interfering RNA (siRNA) constructs for knockdown of FAK or Accell non-targeting siRNA (no. 2) were obtained from Thermo Fisher Scientific (Epsom, UK). The concentration of siRNA constructs was 1 μM in Accell delivery medium containing 2% FCS. The cells were incubated for 5 days (with medium exchange after 3 days), and knockdown efficiency was determined by Western blot analysis.

### Determination of TonEBP/NFAT5 mRNA and protein stability

The cells were incubated in isotonic medium or exposed to hypertonic medium (500 mosmol/kgH_2_O by NaCl addition) for 4 h (mRNA half life) or 24 h (protein half life). Subsequently, actinomycin D (5 μg/ml) and PF-228 (10 μM), or cycloheximide (5 μM) and PF-228 (10 μM), or only vehicle was added. Subsequently, TonEBP/NFAT5 mRNA and protein abundance was determined by qRT-PCR and Western blot analysis, respectively.

### Analysis of TonEBP/NFAT5 nuclear redistribution

Nuclear and cytosolic proteins were isolated using nuclear and cytoplasmic extraction reagent (NE-PER; Pierce) according to the recommendations of the manufacturer with broad specificity protease inhibitor cocktail (Sigma) added at 1:100 (vol/vol). Following the respective treatments, the cells in 60-mm dishes were washed with chilled PBS of equal osmolality as the experimental medium, and the cells were directly harvested by the addition of 200 μl cellular extraction reagent. After centrifugation at 13,000 g for 10 min at 4°C, the supernatant containing cytosolic proteins was saved and the pellet containing nuclear proteins was lysed by addition of 50 μl nuclear extraction reagent. Subsequently, nuclear and cytosolic protein fractions were stored at −80°C until use.

### Presentation of data and statistical analysis

Data are presented as means ± s.e.m. The significance of differences between the means was established using Student's *t*-test when comparing two groups, and analysis of variance (ANOVA) when comparing multiple groups/experimental conditions. *P* < 0.05 was regarded as significant.

## Results

### Intrarenal expression of FAK and activation in response to osmotic stress

**Figure 1:**
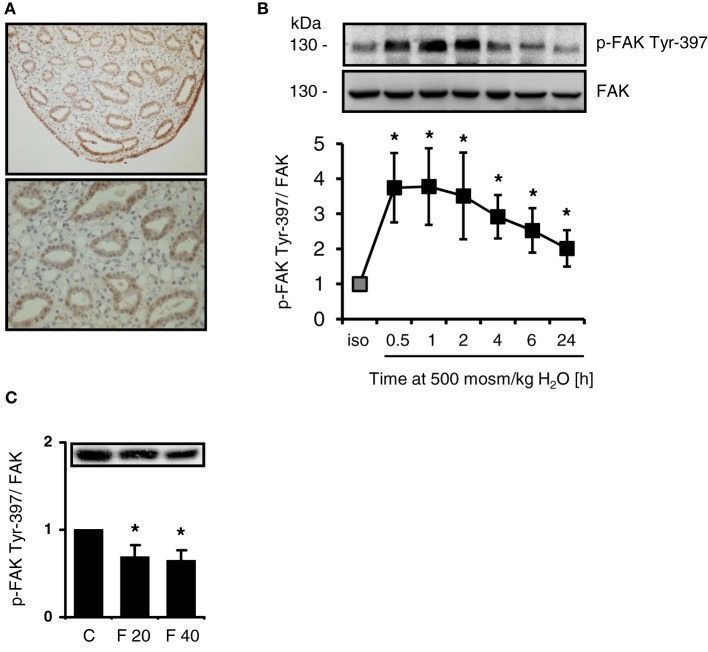
**Intrarenal localization and phosphorylation of FAK in response to osmotic stress. (A)** Sections from normal rat kidney were immunostained for FAK as described in Methods. **(B)** HEK293 cells were incubated in hypertonic medium (500 mosm/kg H_2_O) for the indicated periods. Subsequently, phosphorylation of FAK at Tyr-397 was determined by Western blot analysis and normalized to total FAK abundance. Representative blots are shown. The data are means ± s.e.m. for *n* = 5; ^*^P < 0.05 vs. isotonic control. **(C)** Rats received furosemide (20 mg kg/bw) or PBS (control, *C*) via intravenous injection. After 20 (*F* 20) and 40 (*F* 40) min, FAK Tyr-397 phosphorylation was assessed by Western blot analysis in the renal papilla and normalized to total FAK abundance. Means ± s.e.m. for *n* = 3; ^*^P < 0.05 vs. control.

FAK is expressed abundanty in the cells of the inner medullary collecting duct and in the cells lining the papillary tip (Figure 1A), while staining intensity for FAK gradually decreases from outer medulla to cortex (not shown). Since integrin-mediated activation of FAK causes autophosphorylation at Tyr-397, the phosphorylation status of FAK and FAK abundance was investigated in response to osmotic stress. As shown in Figure 1B, hypertonicity induced rapid and sustained Tyr-397 phosphorylation, which was elevated even 24 h after switching the cells to hypertonic medium. Total FAK abundance was not affected by NaCl addition. To establish whether FAK phosphorylation is responsive to alterations in medullary interstitial tonicity *in vivo*, rats were injected with furosemide to reduce interstitial osmolality in the renal medulla. As expected, furosemide treatment induced profound diuresis (not shown), which was associated with significantly reduced FAK Tyr-397 phosphorylation in the renal papilla both 20 and 40 min after furosemide administration (Figure 1C), while total FAK abundance was not affected. Additionally, the short duration of furosemide treatment is unlikely to affect FAK protein expression levels. In the cortex and outer medulla, phosphorylation was unchanged after furosemide treatment (not shown).

### Effect of FAK inhibition on TonEBP/NFAT5 transcriptional activity

**Figure 2:**
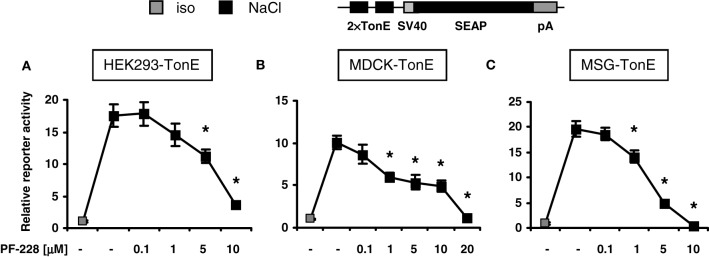
**Effect of FAK inhibition on TonEBP/NFAT5 activity.** HEK293, MDCK, and MSG cells were transfected stably with a TonEBP/NFAT5-driven reporter construct [*HEK293-TonE*, **(A)**; *MDCK-TonE*, **(B)**; *MSG-TonE*, **(C)**]. The cells were incubated in isotonic medium (300 mosm/kg H_2_O) or exposed to hypertonic medium (500 mosm/kg H_2_O by NaCl addition) for 24 h containing the indicated concentrations of PF-228 or only vehicle ethanol. The data are means ± s.e.m. for *n* = 4–8 per time point. ^*^p < 0.05 vs. NaCl + vehicle.

To address the effect of FAK inhibition on TonEBP/NFAT5 activity, three non-related stable TonE reporter cell lines were generated, in which the expression of the reporter gene SEAP is regulated by two TonE motifs. MDCK cells and HEK293 cell are used frequently in experiments addressing the signaling mechanisms during osmoadaptation, however MSG cells, a well-differentiated cell line with characteristics of mesangial cells, are usually not exposed to significant osmotic stress. As demonstrated in Figures 2A–C, addition of the specific FAK inhibitor PF-228, which blocks autophosphorylation at Tyr-397 (Slack-Davis et al., 2007), dose-dependently blunted TonEBP/NFAT5-driven reporter activity in all cell types with a maximal inhibition on a concentration around 10 μM in HEK293 cells. The observation that FAK inhibition diminishes TonEBP/NFAT5 activity in non-related cell lines suggests a conserved regulatory mechanism. There was no evidence of cell death during FAK inhibition at osmolalities ≤500 mosm/kg H_2_O (not shown).

### Effect of FAK inhibition of TonEBP/NFAT5 and target gene expression

**Figure 3:**
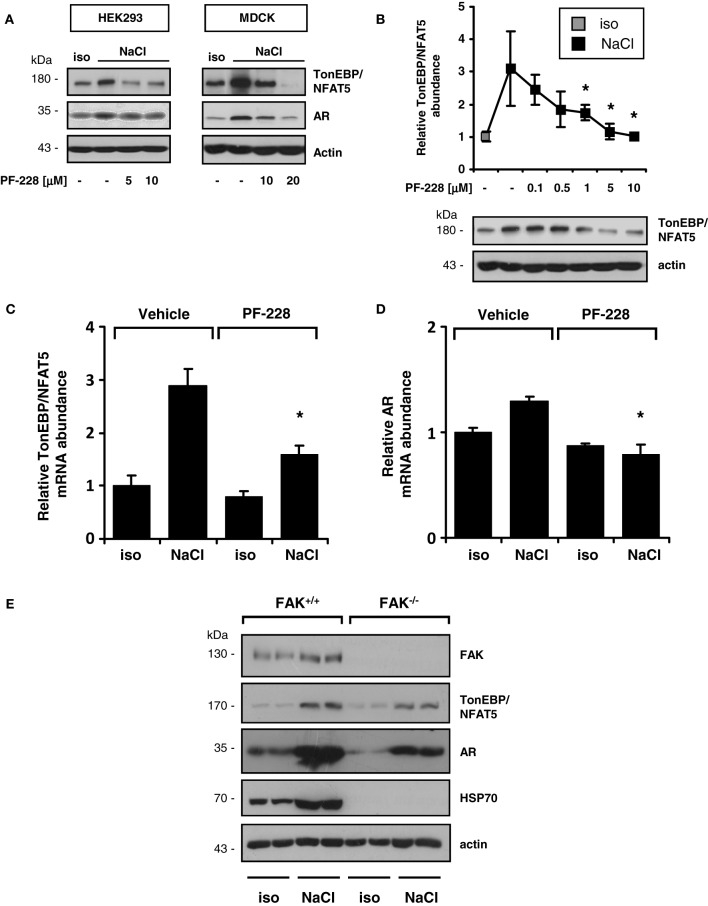
**Effect of FAK inhibition on TonEBP/NFAT5 expression and activity. (A)** HEK293 cells and MDCK cells were incubated under isotonic or hypertonic conditions in the presence of the indicated concentrations of PF-228 or only vehicle ethanol for 24 h. Subsequently, the abundance of TonEBP/NFAT5 and AR was determined by Western blot analysis. Representative blots of three independent experiments are shown. **(B)** TonEBP/NFAT5 abundance was determined in HEK293 cells exposed to the indicated concentrations of PF-228 or vehicle ethanol. The data are means ± s.e.m. for *n* = 4 per time point. ^*^p < 0.05 vs. NaCl + vehicle. **(C,D)** Expression of TonEBP/NFAT5 and AR mRNA in HEK293 cells under the indicated conditions. The data were normalized to actin mRNA abundance. The data are means ± s.e.m. for *n* = 3. ^*^p < 0.05 vs. NaCl + vehicle. **(E)** FAK^+/+^ and FAK^−/−^ MEF were incubated in isotonic medium or exposed to hypertonic medium (450 mosm/kg H_2_O by NaCl addition) for 24 h. Subsequently, the expression of FAK, TonEBP/NFAT5, AR, and HSP70 was determined by Western blot analysis. Representative blots of three independent experiments are shown.

As demonstrated in Figure 3A, FAK inhibition blunted the expression of TonEBP/NFAT5 and the classical target gene aldose reductase (AR) in HEK293 and MDCK cells. To address the contribution of FAK on TonEBP/NFAT5 activation under hypertonic conditions further, HEK293 cells were used for the following experiments. As shown in Figure 3B, inhibition of FAK with PF-228 dose-dependently suppressed TonEBP/NFAT5 induction under hypertonic conditions. According to the findings obtained for protein expression, FAK inhibition was associated with reduced abundance of TonEBP/NFAT5 mRNA and AR mRNA (Figures 3C,D). These findings could be further corroborated in experiments using FAK^−/−^ MEF. Accordingly, MEF lacking FAK not only exhibit substantially reduced expression of TonEBP/NFAT5 but also diminished abundance of AR and HSP70 in response to osmotic stress (Figure 3E).

**Figure 4:**
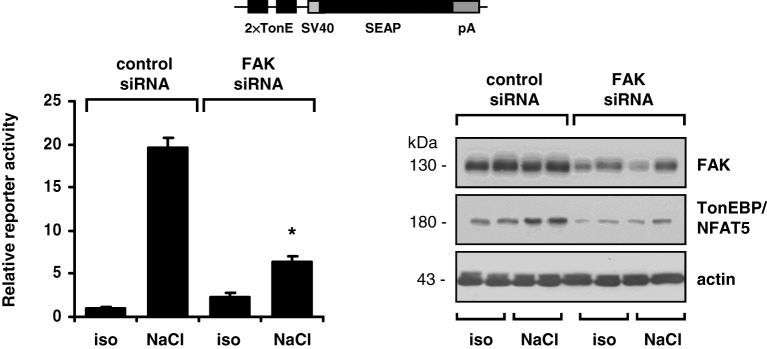
**Effect of FAK knockdown on TonEBP/NFAT5 expression and activity.** HEK293-TonE cells transfected with siRNA specific for FAK or non-targeting control siRNA were incubated for 24 h in isotonic (300 mosm/kg H_2_O) or hypertonic medium (500 mosm/kg H_2_O by NaCl addition). Subsequently, TonEBP/NFAT5-dependent reporter activity was determined as described in Methods. Means ± s.e.m. for *n* = 4–8. ^*^p < 0.05 vs. NaCl + control siRNA. Representative immunoblots for FAK and TonEBP/NFAT5 from four independent experiments are shown.

**Figure 5:**
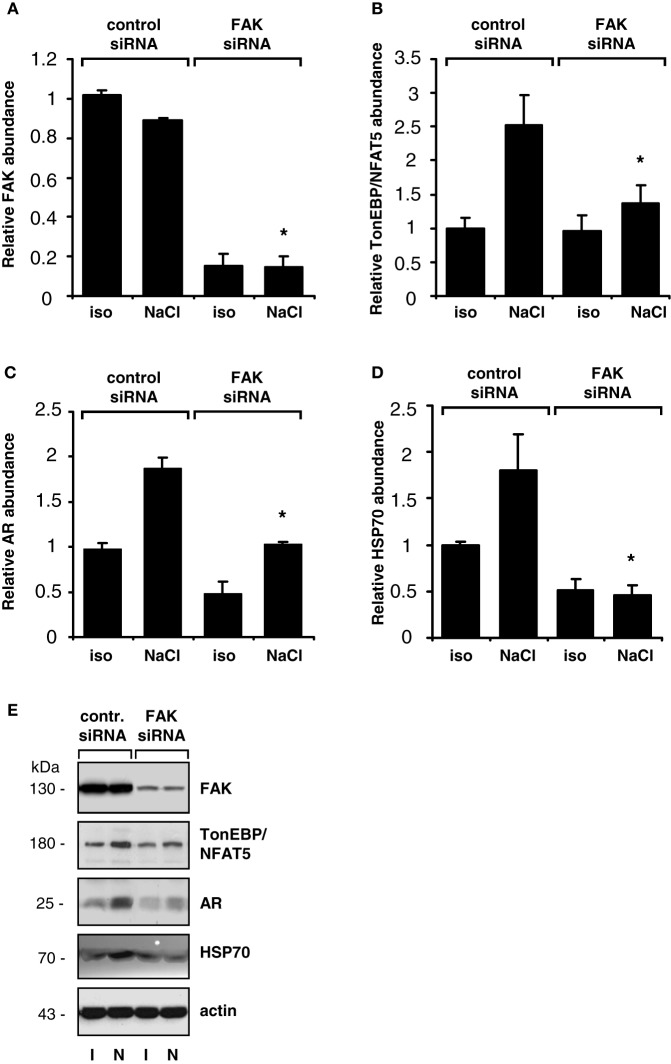
**Effect of FAK knockdown on TonEBP/NFAT5 and target gene expression.** HEK293 cells transfected with siRNA specific for FAK or non-targeting control siRNA were incubated for 24 h in isotonic (300 mosm/kg H_2_O) or hypertonic medium (500 mosm/kg H_2_O by NaCl addition). Subsequently, the abundance of FAK **(A)**, TonEBP/NFAT5 **(B)**, AR **(C)** and HSP70 **(D)** was determined by Western blot analysis. Means ± s.e.m. for *n* = 4. ^*^p < 0.05 vs. NaCl + control siRNA **(E)**. Representative blots are shown.

The findings obtained with pharmacological inhibition of FAK could be reproduced in experiments with siRNA-mediated knockdown of FAK. As shown in Figure 4, in HEK293-TonE cells transfected with specific FAK siRNA, the expression of FAK was diminished by 80–90%, which correlated with strongly reduced TonEBP/NFAT5-dependent reporter activity (Figure 4). As demonstrated in Figure 5, knockdown of FAK reduced TonEBP/NFAT5 abundance by ~50% and largely prevented tonicity-induced upregulation of the classical TonEBP/NFAT5 target genes AR and HSP70.

### FAK inhibition does not affect nuclear redistribution or transactivating activity of TonEBP/NFAT5

**Figure 6:**
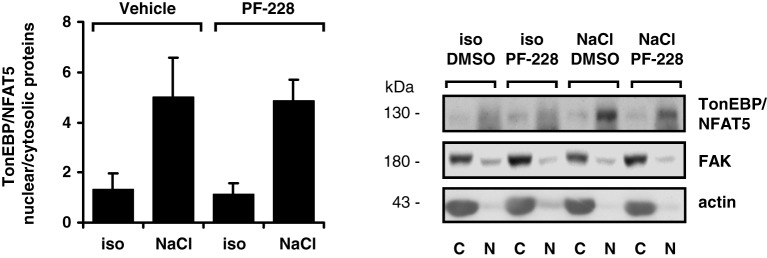
**Effect of FAK inhibition on nuclear translocation of TonEBP/NFAT5.** HEK293 cells were incubated for 4 h in isotonic (300 mosm/kg H_2_O) or hypertonic medium (450 mosm/kg H_2_O by NaCl addition) in the presence or absence of 10 μM PF-228 or only vehicle ethanol. Thereafter, nuclear (*N*) and cytosolic (*C*) protein fractions were analyzed for TonEBP/NFAT5 abundance. Means ± s.e.m. for *n* = 4; Representative Western blots are shown.

**Figure 7:**
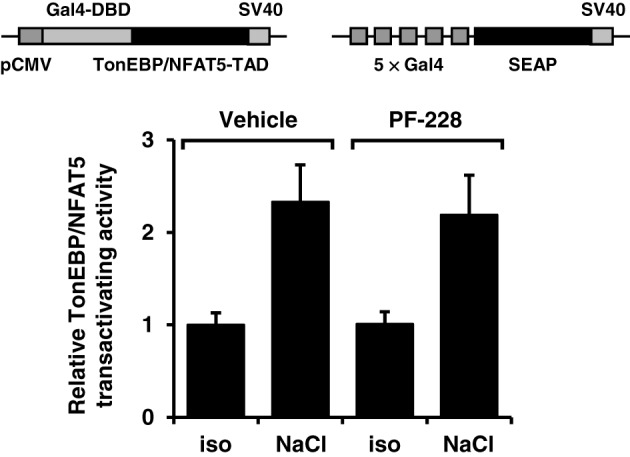
**Effect of FAK inhibition on TonEBP/NFAT5 transactivating activity.** HEK293 cells were electroporated with pGAL4-TonEBP-TAD and pFR-SEAP as described in Methods. Subsequently, the cells were incubated for 24 h in isotonic (300 mosm/kg H_2_O) or hypertonic medium (450 mosm/kg H_2_O by NaCl addition) in the presence or absence of 10 μM PF-228 or only vehicle ethanol. Thereafter, reporter activity was determined as described in Methods. Means ± s.e.m. for *n* = 4–8.

To exclude the possibility that impaired nuclear redistribution of TonEBP/NFAT5 contributes to reduced expression of TonEBP/NFAT5 target genes under hypertonic conditions during FAK inhibition, TonEBP/NFAT5 abundance was assessed in nuclear and cytosolic protein fractions. As shown in Figure 6, osmotic stress caused substantial accumulation of TonEBP/NFAT5 in the nuclear protein fraction, which was however insensitive to FAK inhibition. A further major mechanism for induction of target genes is increased transactivating activity of TonEBP/NFAT5, which was however not affected by inhibition of FAK (Figure 7).

### FAK inhibition reduces TonEBP/NFAT5 mRNA stability

**Figure 8:**
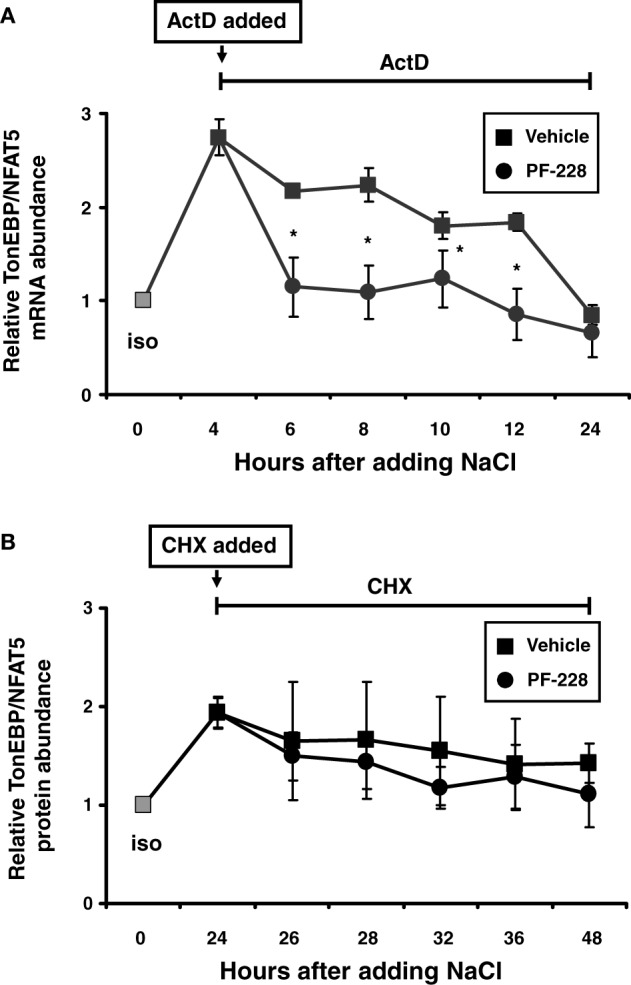
**Effect of FAK inhibition on TonEBP/NFAT5 mRNA and protein half-life. (A)** HEK293 cells remained in isotonic medium (300 mosm/kg H_2_O) or were incubated in hypertonic medium (500 mosm/kg H_2_O by NaCl addition) for 4 h. Subsequently, actinomycin D (5 μg/ml) and PF-228 (10 μM) or vehicle ethanol was added and TonEBP/NFAT5 mRNA abundance was determined at the indicated time points by qRT-PCR. **(B)** The cells remained in isotonic medium or were incubated in hypertonic medium for 24 h prior to the addition of cycloheximide (5 μM) and PF-228 (10 μM) or vehicle ethanol. Thereafter, TonEBP/NFAT5 protein abundance was determined at the indicated time points by Western blot analysis. The data are means ± s.e.m. for *n* = 4 per time point. ^*^p < 0.05 for NaCl + vehicle vs. NaCl + PF-228.

Since stabilization of the TonEBP/NFAT5 mRNA has been reported under hypertonic conditions (Cai et al., 2005), this phenomenon was reinvestigated to establish whether this process requires FAK activity. HEK293 cells were exposed to hypertonic medium for 4 h to increase TonEBP/NFAT5 mRNA (Figure 8A). To stop transcription, actinomycin D was added 4 h after increasing the medium osmolality in parallel with PF-228, and the rate at which TonEBP/NFAT5 mRNA decreased was determined. As demonstrated in Figure 8A, osmotic stress increased TonEBP/NFAT5 mRNA abundance several fold. The abundance slightly declined thereafter but remained detectable at high levels for 6–8 h without FAK inhibition. Based on the data for mRNA decay, the TonEBP/NFAT5 mRNA half-life under hypertonic conditions was calculated to be ~16.5 h without FAK inhibition (not shown). In the presence of PF-228 however, TonEBP/NFAT5 mRNA abundance was restored to almost isotonic control levels within 2–4 h (Figure 8A), accordingly mRNA half-life was substantially reduced to ~8.9 h (not shown). As expected, FAK phosphorylation was inhibited by PF-228 after increasing the medium osmolality (not shown). Protein stability was determined in parallel experiments using cycloheximide for inhibition of translation. In contrast to mRNA stability, FAK inhibition had no major effect on protein degradation in cells exposed to osmotic stress (Figure 8B).

### FAK inhibition has no effect on TonEBP/NFAT5 promoter activity

**Figure 9:**
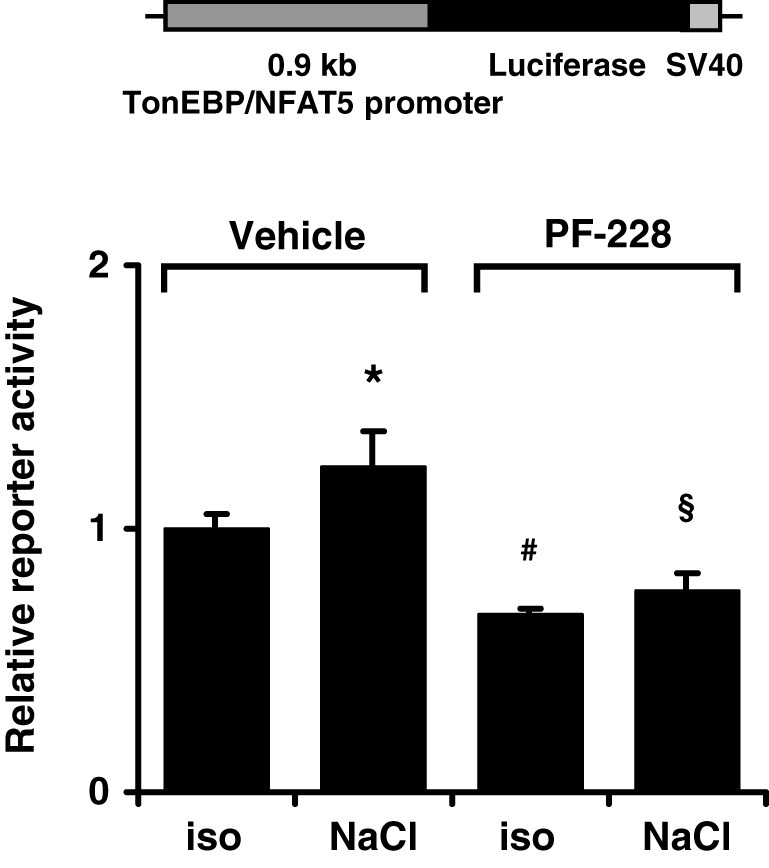
**Effect of FAK inhibition on TonEBP/NFAT5 promoter activity.** HEK293 cells were transfected with a reporter construct containing 0.9 kb of the human TonEBP/NFAT5. Subsequently, the cells were incubated for 24 h in isotonic (300 mosm/kg H_2_O) or hypertonic medium (500 mosm/kg H_2_O by NaCl addition) in the presence of 10 μM PF-228 or only vehicle ethanol. Thereafter, reporter activity was determined as described in Methods. Means ± s.e.m. for *n* = 4 ^*^p < 0.05 vs. iso + vehicle; ^#^*p* < 0.05 vs. iso + vehicle; ^§^*p* < 0.05 vs. Nacl + vehicle.

To characterize the mechanism by which FAK inhibition reduces TonEBP/NFAT5 abundance further, HEK293 cells were transfected with a reporter construct driven by a 0.9-kb fragment of the human TonEBP/NFAT5 promoter. As demonstrated in Figure 9, reporter activity only increased slightly under hypertonic conditions, suggesting that increased transcription of the TonEBP/NFAT5 makes no major contribution to upregulation of TonEBP/NFAT5 in response to osmotic stress. Nevertheless, inhibition of FAK with PF-228 slightly reduced reporter activity both under isotonic and hypertonic conditions. However, we cannot exclude the contribution of regulatory elements upstream of the 0.9 kb promoter sequence, which is a limitation of the present study. Nevertheless, in most reporter studies, promoter fragments of ~1 kb upstream of the transcription start site are regarded as suitable.

### FAK inhibition diminishes the activity of the TonEBP/NFAT5 3′-UTR

**Figure 10:**
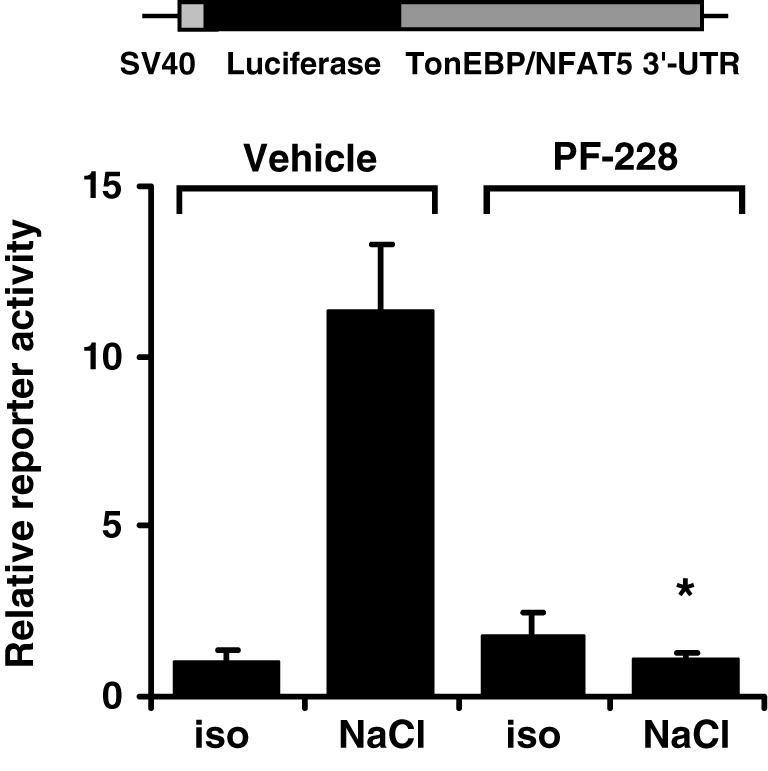
**Effect of FAK inhibition on TonEBP/NFAT5 3′-UTR activity.** HEK293 cells were transfected with a reporter construct containing the full-length TonEBP/NFAT5 3′-UTR distal to the luciferase ORF. Subsequently, the cells were incubated for 24 h in isotonic (300 mosm/kg H_2_O) or hypertonic medium (450 mosm/kg H_2_O by NaCl addition) of 10 μM PF-228 or only vehicle ethanol. Thereafter, reporter activity was determined as described in Methods. Means ± s.e.m. for *n* = 4 ^*^p < 0.05 vs. NaCl + vehicle.

The TonEBP/NFAT5 3′-UTR contains several putative binding sites for miRNA. The latter are important regulators of gene expression by targeting the 5′-UTR of mRNAs to induce mRNA cleavage and/or translational repression. Since FAK inhibition reduces mRNA stability, the activity of the TonEBP/NFAT5 3′-UTR was determined in HEK293 cells transfected with a reporter construct in which the luciferase ORF was fused to the full-length 8.3-kb TonEBP/NFAT5 3′-UTR. As demonstrated in Figure 10, reporter activity increased dramatically under hypertonic conditions, and this increase was completely abolished by FAK inhibition. These observations indicate that the TonEBP/NFAT5 3′-UTR is strongly sensitive to changes in tonicity, and that this process requires FAK activity.

## Discussion

FAK transduces mechanical forces, such as are present during cell shrinkage in cells exposed to osmotic stress, into intracellular signals to elicit adaptive cellular responses (Hauck et al., 2002; Mitra and Schlaepfer, 2006). FAK is expressed abundantly in the renal medulla and is activated by autophosphorylation at Tyr-397 under hypertonic conditions (Figures 1A,B). Notably, elevated levels of phospho-Tyr-397 are detectable even 24 h after the onset of osmotic stress and phosphorylated FAK is detectable in collecting ducts of rats with free access to water (Holzapfel et al., 2007). Moreover, Tyr-397 phosphorylation is reduced in furosemide-treated rats (Figure 1C). Given these findings, FAK would appear to meet the requirements for osmosensing and signaling to TonEBP/NFAT5. The classical stimulus for FAK activation is integrin engagement/clusterin in response to alterations in contact to ECM components (Mitra et al., 2005). Interestingly, cells lacking integrin α1β1 show impaired induction of TonEBP/NFAT5 and osmoprotective genes under hypertonic conditions (Moeckel et al., 2006). Accordingly, mice deficient for integrin α1 have impaired ability to accumulate organic osmolytes in the inner medulla due to reduced induction of osmolyte transporters and AR (Moeckel et al., 2006).

The activity of TonEBP/NFAT5 is regulated at multiple levels (summarized in Neuhofer and Beck, 2005; Jeon et al., 2006). It is well established that hypertonicity entails nuclear translocation, increased transactivation and elevated expression of TonEBP/NFAT5. Either pharmacological inhibition of FAK or siRNA-mediated knockdown drastically reduced TonEBP/NFAT5 expression and activity. However, FAK inhibition had no effect on nuclear translocation or on transactivating activity of TonEBP/NFAT5, which are classical mechanisms of activation. Nevertheless, TonEBP/NFAT5 mRNA and protein abundance were diminished by ~50%, suggesting that FAK regulates TonEBP/NFAT5 expression. Using a reporter construct containing 0.9 kb of the human TonEBP/NFAT5 promoter, hypertonicity achieved by adding NaCl slightly, but significantly, increased reporter activity compared with that in isotonic controls. Inhibition of FAK reduced reporter activity significantly under isotonic and hypertonic conditions. To our knowledge, this is the first study addressing TonEBP/NFAT5 promoter activitiy. However, the minor increase in reporter activity in cells exposed to osmotic stress argues against the notion that stimulation of TonEBP/NFAT5 transcription is a major mechanism accounting for the increase in TonEBP/NFAT5 mRNA and protein abundance.

Inhibition of FAK, however, substantially reduced TonEBP/NFAT5 mRNA stability and abolished reporter activity in a reporter construct in which the luciferase ORF is fused to the TonEBP/NFAT5 3′-UTR, and hence reflects the stabilizing effects of the 3′-UTR. These findings are in agreement with results of Cai et al. demonstrating stabilization of the TonEBP/NFAT5 mRNA under hypertonic conditions (Cai et al., 2005). Bioinformatics analysis revealed several miRNA recognition elements (MRE) within the TonEBP/NFAT5 3′-UTR. In recent years, miRNA have emerged as crucial regulators of gene expression at the post-transcriptional level (Chekulaeva and Filipowicz, 2009). Interestingly, the expression of specific miRNAs is highly responsive to alterations in ambient tonicity. In IMCD3 cells, hypertonicity down-regulates the expression of miR-200b and miR-717 by 80% within 2 h after exposure to osmotic stress and the expression of these miRNAs in the renal papilla correlates negatively with urine osmolality and the expression of TonEBP/NFAT5 in this kidney zone (Huang et al., 2011). In agreement with these observations, overexpression of miR-200b and miR-717 significantly reduces mRNA and protein expression of TonEBP/NFAT5 and its transcriptional activity (Huang et al., 2011). Whether FAK is involved in the regulation of specific miRNAs is currently not known. Based on mRNA decay experiments, the half-life of the TonEBP/NFAT5 mRNA was calculated to be about 8.9 h with FAK inhibition and ~16.5 h in vehicle-treated cells. These observations are in good agreement with data from Cai et al., who reported a half-life of ~6 h under isotonic conditions (Cai et al., 2005). Although mRNA half-life was not determined under hypertonic conditions, those authors noted that the mRNA was not, or only slightly, degraded in the initial 4–6 h after the onset of osmotic stress (Cai et al., 2005). These findings parallel the observations in the present study that also demonstrate stabilization of the TonEBP/NFAT5 mRNA at high levels after adding NaCl. Inhibition of FAK however restores the mRNA abundance close to isotonic control levels within 2 h (Figure 8A). These observations and the only marginal induction of TonEBP/NFAT5 promoter activity in response to osmotic stress support the notion that mRNA stabilization is a major mechanism that accounts for increased TonEBP/NFAT5 abundance under hypertonic conditions.

In conclusion, FAK is abundant in the renal papilla and is activated by hypertonicity *in vitro* and *in vivo*. FAK activity in turn is required for increasing the abundance of TonEBP/NFAT5 by stabilizing its mRNA, which depends on the presence of the TonEBP/NFAT5 3′-UTR. Hence, tonicity-mediated, FAK-dependent stabilization of the mRNA appears to be the major mechanism by which the abundance and activity of TonEBP/NFAT5 increases in response to osmotic stress.

### Conflict of interest statement

The authors declare that the research was conducted in the absence of any commercial or financial relationships that could be construed as a potential conflict of interest.
