# Supplementary material for: Focal adhesion kinase regulates the activity of the osmosensitive transcription factor TonEBP/NFAT5 under hypertonic conditions
Source: Front Physiol. 2014 Apr 4;5:123. doi: 10.3389/fphys.2014.00123 (PMC3983490; doi:10.3389/fphys.2014.00123)
Supplement: Supplementary file 1 [file Presentation1.PDF]

Supplementary Figure 1

TonEBP/NFAT5 promoter sequence used in reporter assays

AGGTGGATTCACATGATCCAATTTTTTAATTGTCTTCTATGTCCCACTCC

GAATAACCCGGATGCCCCAGCACAATCAGAGAGATCGTTTTTTTTAAAAA

AAATTTCAGCCTCCAAAGGTAGGAGGGGAGTGTTAGGGGAGAAAGTACTT

TAATTAAAAATCAATAACTCGAGGTTTTTGGGATACGGTTTGCCATTTCC

TAATTAAGAAATGGGTTTGACAGTCCCTTTGTACACACTGCTATGCAAAC

CCCAAAGGGTTGGCGGCTGTCCGGGCGATGACACTCCGGTCCCCTGCGAG

ACCCCGGGCCAGCCAGGCCCGTCCGCCGCCGGCCTCTGGGGTCCGTCCCC

GGCTCGCGCAGACCTCTCGCTTCTCTCGGCTCTGTCTCCTGCGCTCAGCT

CTGCTCGGGGCCGGCCGCCTCAGGCTCGCCGCCACCAGGTCGTTGCAAAT

ACCTTTTCCCTCCCCGGGGCCCCAGCGCGCGGCCACCTCCCAGCCTCCCC

CCCTCCCACCCTGGCAGCGGGGCCCTTTCCCGGCTCAGGAACAGCAGCAG

CCCGGGCCGCGCCGGCAGGAAGCGAGGCCCATGTTGCTGCTGTTCCCTGG

CGCGCCTCCCCGCCCTCCGGGGGCCGCCACGGCTCTTCCGCGCTCCCGGG

CACCCCCCTCCGCGCCTGCGCTGTGCCCCACGGGGGCGGGGCTCAGATTC

CTGTCAGCGGCGGCGGCGGTGGCGGCGACCGTCAGTTTTCGCTGAGGAGA

AACACGAAACGGACCCTTTGGCTCTCCCCCTTCCCCTTCCCCGTCCTGAA

CCCCTCTCCTGGTCACCGAGAATCAGTCCCCGTGGAGTTCCCCCTCCACC

TCGCCATCGTTTCCTCGGTCCTCGGCCCAGTGGAAGTCACTACCCTCGAG

GAGGAG
